# Validation of the Fetal Lamb Model of Spina Bifida

**DOI:** 10.1038/s41598-019-45819-3

**Published:** 2019-06-27

**Authors:** Luc Joyeux, Alexander C. Engels, Johannes Van Der Merwe, Michael Aertsen, Premal A. Patel, Marjolijn Deprez, Ahmad Khatoun, Savitree Pranpanus, Marina Gabriela Monteiro Carvalho Mori da Cunha, Stephanie De Vleeschauwer, Johanna Parra, Katerina Apelt, Myles Mc Laughlin, Frank Van Calenbergh, Enrico Radaelli, Jan Deprest

**Affiliations:** 1 Department of Development and Regeneration, Cluster Urogenital, Abdominal and Plastic surgery, Biomedical Sciences, Katholieke Universiteit (KU) Leuven, Leuven, Belgium; 2 Center for Surgical Technologies, Faculty of Medicine, KU Leuven, Leuven, Belgium; 3 Department of Obstetrics and Gynecology, University Hospital Bonn, Bonn, Germany; 4 Department of Obstetrics and Gynecology, Division Woman and Child, Fetal Medicine Unit, University Hospital Gasthuisberg, Leuven, Belgium; 5 Department of Radiology, University Hospital Gasthuisberg, Leuven, Belgium; 6 Radiology Department, Great Ormond Street Hospital for Children, NHS Foundation Trust, London, United Kingdom; 7 Research group Experimental Neurosurgery and Neuroanatomy, Department of Neurosciences, KU Leuven, Leuven, Belgium; 8 Experimental Otorhinolaryngology, Department of Neurosciences, KU Leuven, Leuven, Belgium; 9 Department of Obstetrics and Gynecology, Faculty of Medicine, Prince of Songkla University, Hat Yai, Songkhla, Thailand; 10 Animal Research Center, Biomedical Sciences, KU Leuven, Leuven, Belgium; 11 Barcelona Center for Maternal Fetal and Neonatal Medicine, University of Barcelona, Barcelona, Spain; 12 Department of Neurosurgery, University Hospital Gasthuisberg, Leuven, Belgium; 13 Department of Pathobiology, Ryan Veterinary Hospital, University of Pennsylvania School of Veterinary Medicine, Philadelphia, PA United States; 14 Institute of Women’s Health, University College London Hospitals, London, United Kingdom

**Keywords:** Developmental disorders, Disease model, Experimental models of disease, Neurological disorders, Pathogenesis

## Abstract

A randomized trial demonstrated that fetal spina bifida (SB) repair is safe and effective yet invasive. New less invasive techniques are proposed but are not supported by adequate experimental studies. A validated animal model is needed to bridge the translational gap to the clinic and should mimic the human condition. Introducing a standardized method, we comprehensively and reliably characterize the SB phenotype in two lamb surgical models with and without myelotomy as compared to normal lambs. Hindbrain herniation measured on brain magnetic resonance imaging (MRI) was the primary outcome. Secondary outcomes included gross examination with cerebrospinal fluid (CSF) leakage test, neurological examination with locomotor assessment, whole-body MRI, motor and somatosensory evoked potentials; brain, spinal cord, hindlimb muscles, bladder and rectum histology and/or immunohistochemistry. We show that the myelotomy model best phenocopies the anatomy, etiopathophysiology and symptomatology of non-cystic SB. This encompasses hindbrain herniation, ventriculomegaly, posterior fossa anomalies, loss of brain neurons; lumbar CSF leakage, hindlimb somatosensory-motor deficit with absence of motor and somatosensory evoked potentials due to loss of spinal cord neurons, astroglial cells and myelin; urinary incontinence. This model obtains the highest validity score for SB animal models and is adequate to assess the efficacy of novel fetal therapies.

## Introduction

Spina bifida is the most frequent congenital anomaly of the central nervous system accounting for 4.9/10,000 live births in Europe and 3.17/10,000 in the United States^1–4^. The most common clinical presentation is an open form called spina bifida aperta (SBA). SBA is a non-lethal yet chronic and progressive disease with significant neurological morbidity, its severity largely depending on the anatomical level of the defect^5–7^. The pathogenesis of this condition is explained by two consecutive hits^8–14^. This malformation arises when the neural tube fails to close by the 6^th^ week of gestation. This exposes the neural elements continuously to direct trauma and neurotoxic agents in the amniotic fluid during gestation, leading to progressive changes. Over the lesion there is leakage of cerebrospinal fluid (CSF)^15–17^, leading to a suction gradient, in turn causing hindbrain herniation and smaller posterior fossa and cerebellum (both referred to as Chiari II malformation) and/or ventriculomegaly^9,15,18–20^. There is also progressive limb function loss, and, in some fetuses, development of club feet and/or kyphosis. After birth, children display various degrees of extremity paraparesis and paralysis, orthopedic deformation of spine and limbs, as well as bladder, bowel and sexual dysfunction^5^. In addition, children with SBA almost invariably have hindbrain herniation^21^ and variable degrees of ventriculomegaly that may require CSF shunting when symptomatic^5^. This progressive prenatal deterioration paved the way for fetal surgery. A randomized clinical trial demonstrated that fetal repair of SBA provides better neurologic outcomes than postnatal repair, yet it still requires general anesthesia, laparotomy and large hysterotomy^12^. Therefore, new less invasive techniques are being proposed, yet they are not supported by sufficient experimental studies^22,23^.

The use of animals to model human diseases in biomedical research relies on the notion that basic processes are sufficiently similar across species to allow extrapolation, and therefore clinical translation of new treatments. International FDA and EMA guidelines have described the key characteristics of an appropriate animal model^24–26^: the modeled disease should mimic the human condition both in etiology, pathophysiology, symptomatology and response to therapeutic interventions. Subsequently, it can be used to provide substantial evidence of efficacy of novel and ethically acceptable treatments. A scoring system has been developed to describe the degree of validity of a disease model using five parameters, i.e. (1) the species used, (2) its complexity, ranging from molecules and cells to animals, (3) the extent of simulation of the anatomy and pathophysiology of the disease, (4) the face validity, i.e. how it reproduces symptomatology and (5) the predictivity, meaning in this context the degree of response to treatment (Table S1)^27–29^.

Taken together, the above-quoted evidence highlights the relevance of a validated SBA experimental model in translational research^23,30^. There has indeed been a huge interest in the creation of appropriate animal models, ranging from cells^31^ to rodents^32–34^ to large models, including the sheep^23^. Given that SBA is an embryologic defect with the occurrence of progressive *in utero* deterioration, embryonic and fetal models have been developed. However small animal models are not suitable to evaluate feasibility, safety and efficacy of any fetal therapy due to their small size and the different physiology. Therefore, large models are required before new therapies are clinically implemented. The most frequently used large animal model is the fetal lamb^23^. This is a surgical model where the lesion is induced at midgestation by skin, paraspinal muscle, spinal lamina and dura resection. That intervention simulates the morphologic and functional consequences of spinal cord damage with somatosensory dysfunction at birth^10,35^. In later studies, it was shown that adding a longitudinal myelotomy induces hindbrain herniation, typical for this condition, yet the spinal consequences were not simultaneously documented^11,16^. Herein we determine the validity score of these two most frequently used lamb models. For that purpose, we introduce a standardized method to comprehensively and reliably characterize the full phenotype of SBA.

## Methods

This experiment was approved by the Ethical Committee on Animal Experimentation of the Faculty of Medicine (P285-2014). It followed the NC3Rs (National Center for the Replacement, Refinement, and Reduction of Animals in Research) and the EQUATOR and ARRIVE (Animals in Research Reporting *In Vivo* Experiments) guidelines for animal research^26,36^.

### Study design

As we aimed to develop a method to comprehensively and reliably characterize the SBA phenotype in the lamb, our study required three groups: non-operated fetal lambs (negative controls; normal group) and SBA lambs with (myelotomy group) or without (non-myelotomy group) longitudinal myelotomy. The goal was to determine which group best reproduced the lumbar SBA phenotype at birth^5^, i.e. a combination of hindbrain herniation (primary endpoint determined on brain Magnetic Resonance Imaging (MRI), used in the power calculation), ventriculomegaly, lumbar CSF leakage through the scar, hindlimb somatosensory motor deficit on clinical examination and Motor Evoked Potentials (MEP) and Somatosensory Evoked Potentials (SEP) recordings, urinary incontinence, MRI and histomorphometric changes in the brain, spinal cord, hindlimb muscles, bladder and rectum. Each of these received a weighted score.

Sample size was based on two-sided Fisher’s exact test with 5% significance level, 80% power to detect a difference between 92%^11,16,23^ and 0% in hindbrain herniation as on neonatal MRI, i.e. 6 fetuses alive at birth per group. Fetal lambs were assigned first to the non-myelotomy and then to myelotomy group chronologically to ensure homogeneity. Littermates were assigned to the control group, and because not all were multiplets, additional ewes were included.

### Experimental procedures

**Figure 1:**
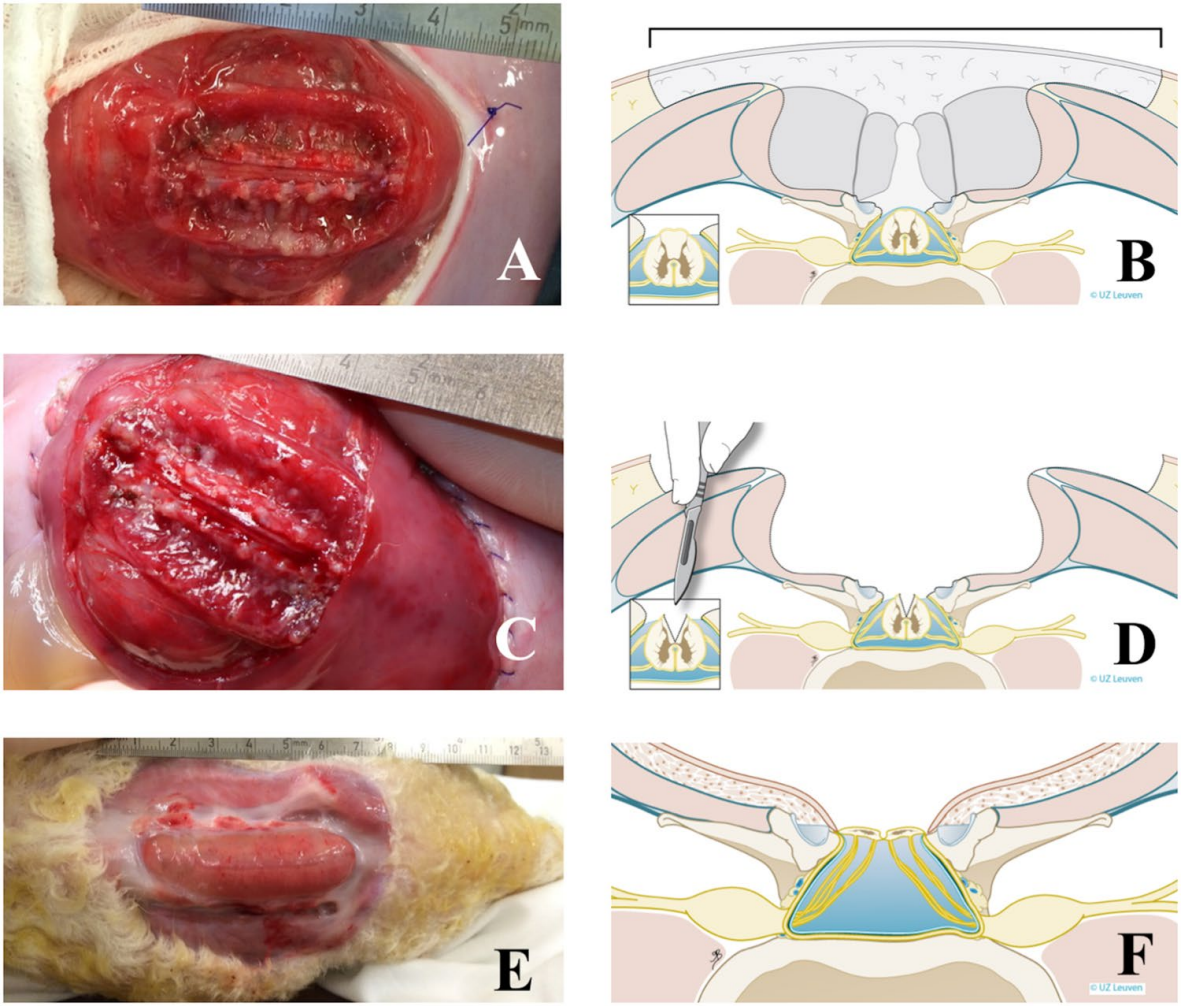
Technique of SBA induction. (**A**) Induction of the lesion at 75 days of gestation (term, 145 days), consisting of a ≥4 cm long skin and myofascial resection, ≥5‐level laminectomy, and ≥5‐level durectomy. In this case, without ≥5‐level myelotomy. (**B**) Artist impression of (**A**). (**C**) Same as (**A**), yet with ≥5‐level myelotomy, i.e., posterior mid-sagittal incision of the spinal canal. (**D**) Artist impression of (**C**). (**E**) Lesion as above in (**C**), at birth (143 days). (**F**) Artist impression of (**E**). Drawings by Myrthe Boymans (www.myrtheboymans.nl) for and copyright by UZ Leuven, Belgium. (SBA, spina bifida aperta).

Time-dated pregnant Swifter sheep were provided by the university farm. Following sedation with intra-muscular xylazine injection (0.3 mg/Kg XYL-M 2%, V.M.D. s.a., Arendonk, Belgium), general anesthesia was induced with intravenous propofol (4 mg/Kg Propovet Multidose 10 mg/mL, Abbot, Breda, The Netherlands) and maintained with 1.5–2.5% isoflurane (Iso-Vet 1000 mg/g, Dechra, Northwich, UK) in 100% oxygen (5 L/min). Immediately before the incision, the sheep was injected intravenously 1.5 g of cefazolin (Cefazolin Sandoz 1 g, Sandoz Novartis, Holzkirchen, Germany) and 0.02 mg/Kg of buprenorphine (Vetergesic multidose 0.3 mg/mL 10 mL, Ecuphar, Oostkamp, Belgium). The uterus was exposed through a parasagittal midline laparotomy, following which a 6–8 cm sutured (PDS3/0, Ethicon, Somerville, NJ, United States) hysterotomy was made to expose the lumbar region of the fetus. Following an intramuscular injection of 0.01 mg/kg of fentanyl (Fentanyl Janssen 0.05 mg/mL, 10 mL ampoules, Janssen-Cilag, Beerse, Belgium), a SBA lesion was induced at around 75 days of gestation as follows. First a ≥4 cm long skin and fascial defect was created over the lumbar spine from the tip of the lower rib to the posterior iliac crest. Second a bilateral paraspinal muscle resection was made. Third a ≥5-level lumbar laminectomy from L1 to L5–6 was performed, followed by durectomy^23,37^. In an additional group, the above was completed by mid-sagittal posterior longitudinal myelotomy^11,23^ (Fig. 1).

Thereafter, the fetus was returned to the uterus, the amniotic fluid replaced with Hartmann solution (Hartmann 1000 mL, Baxter Healthcare, Deerfield, IL, United States) until a vertical pocket of ≥30 mm on ultrasound and mixed with 0.5 g of cefazolin. The hysterotomy was closed with PDS 3/0 (Ethicon) and the laparotomy with PDS 0 (Ethicon) for the linea alba and Monocryl 2/0 (Ethicon) for the subcutaneous fascia and the skin. The ewe was given a tocolytic, medroxyprogesterone acetate (Depo-Provera 150 mg in one mL, Pfizer, NYC, NY, United States) and 10 mL of local anesthetics at the scar (Lignocaine HCl 2% adrenaline, Kela, Hoogstraten, Belgium). When the animal was back in her stable standing and eating, she was administered 0.01 mg/kg of fentanyl as postoperative analgesia.

All lambs were delivered by cesarean section through flank incision around term (145 days of gestation) under spinal anesthesia as described earlier^38^. On day two of life, general anesthesia was induced using intravenous propofol. An initial bolus of 5 mg/Kg was given, with additional boluses of half a dose as necessary to abolish palpebral reflexes. At the same time, systemic hydration was maintained with boluses of 2 mL of an isotonic solution to keep the animal hemodynamically stable. Firstly, the lambs underwent whole-body MRI for around 60 min. Secondly, MEP and SEP were recorded and finally the lambs were euthanized with an overdose of propofol and histological samples were harvested.

### Outcome measures

All outcomes data were collected step-by-step by two independent observers and analyzed twice by at least two independent raters, all blinded to the allocated experimental condition (Table S2).

#### Gross examination

**Figure 2:**
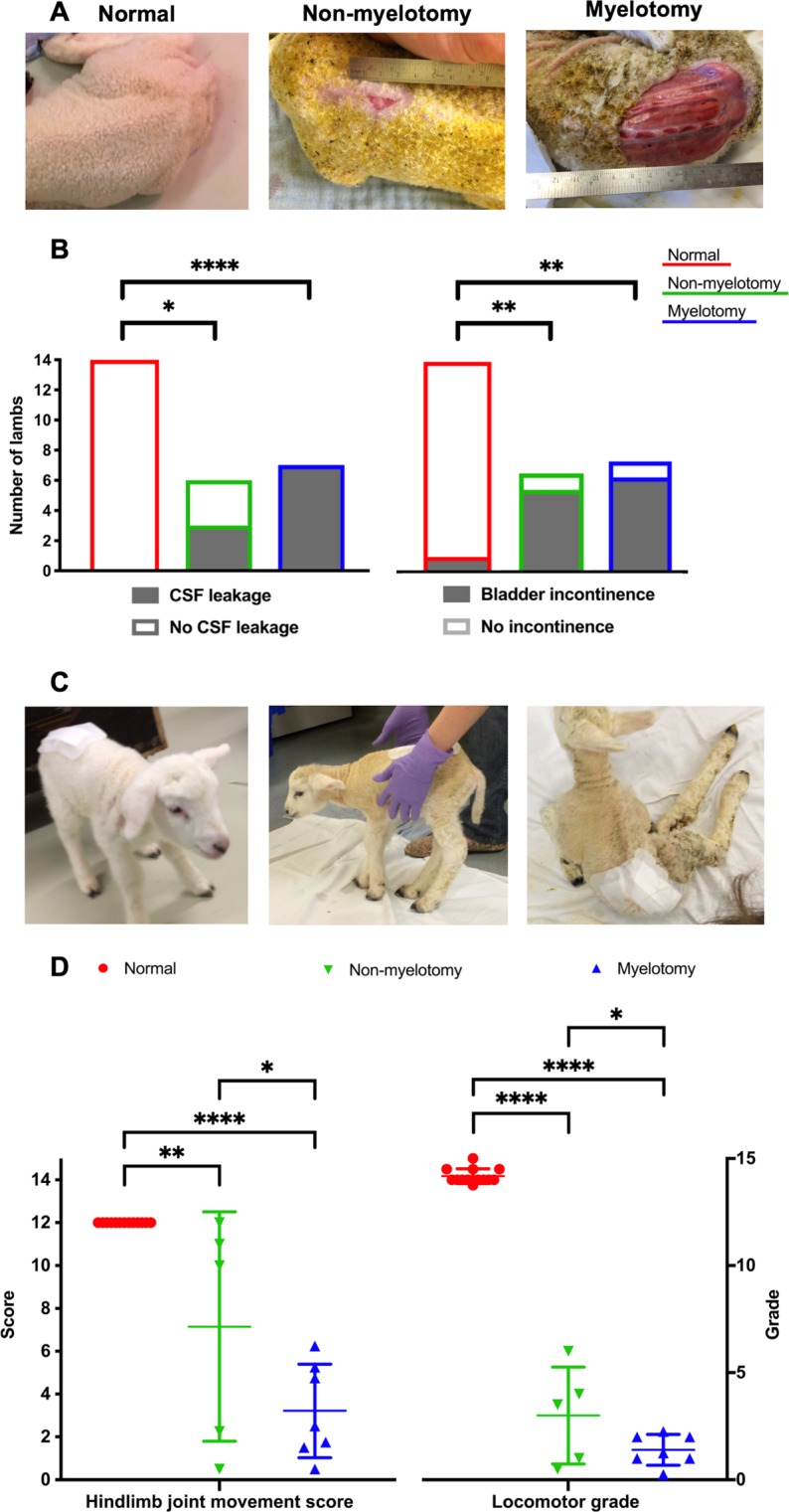
Assessment of hindlimb neuromotor deficit in neonatal lambs. We used the 12-point joint movement score and the 15-point motor grade of the sheep locomotor rating scale. (**A**) lumbar region with or without skin defect; (**B**) results of the cerebrospinal fluid (CSF) leakage and bladder incontinence; (**C**) locomotor deficit; (**D**) results of the joint score and the motor grade per group. [Significance as *0.05 ≥ p > 0.01; **0.01 ≥ p > 0.001; ***0.001 ≥ p > 0.0001; ****p ≤ 0.0001].

After cleaning and drying the lambs, we measured the size of the lumbar defect (skin and neural placode separately). Then we applied a blotting paper (Easy V2 White, 863048, Lucart Professional, Diecimo, Italy) above it to objectify a skin lumbar CSF leakage (Fig. 2A).

#### Neurological clinical examination

After applying a bandage over the lumbar region to blind the assessor, neonatal lambs were assessed just prior to general anesthesia. We used a standardized neurological clinical examination protocol for large animals as earlier described and using reproducible variables, i.e. abnormal spontaneous head movement (tremor or spasm), abnormal gait (paraparesis or paraplegia), urinary incontinence, lumbar-sacral sensory deficit and hindlimb movements^39^. Urinary incontinence was defined as leakage when the lamb was lifted gently upwards with one hand over the bladder, hence creating bladder pressure by its own weight. It was determined independently by two observers. In case of disagreement, both observers performed a third examination to reach a consensus. Videos to document the hindlimb movements were made and off line assessed by two board-certified veterinarians to quantify a hindlimb joint movement score and a locomotor grade from the sheep locomotor rating scale earlier validated in neonatal lambs (Fig. 2C)^40^. In total 6 outcomes were assessed (Table S2).

#### Whole-body MRI

Under general anesthesia, we performed a 3 Tesla MRI scanner (MAGNETOM Prisma, Siemens Healthcare, Erlangen, Germany) with a 15-channel phased-array knee coil for the brain and an 18-channel body coil for the body respectively. T2-weighted sequences of the brain were primarily acquired in three planes (Table S3), the transverse plane being defined as a line perpendicular to the line joining the anterior and posterior lower parts of the corpus callosum on the mid-sagittal plane. We used anatomical T2- and T1-weighted sequences of the brain to identify subacute or old ischemic or hemorrhagic areas. The body was imaged with T2-weighted sequences in the sagittal plane to evaluate the spinal cord, kidneys and bladder. Additionally, axial plane sequences were acquired for detailed evaluation of the spinal defect. The hindlimb muscles were evaluated using 3D T1-weighted images that could be reconstructed in multiple planes following the short axis of both hindlimbs.

**Figure 3:**
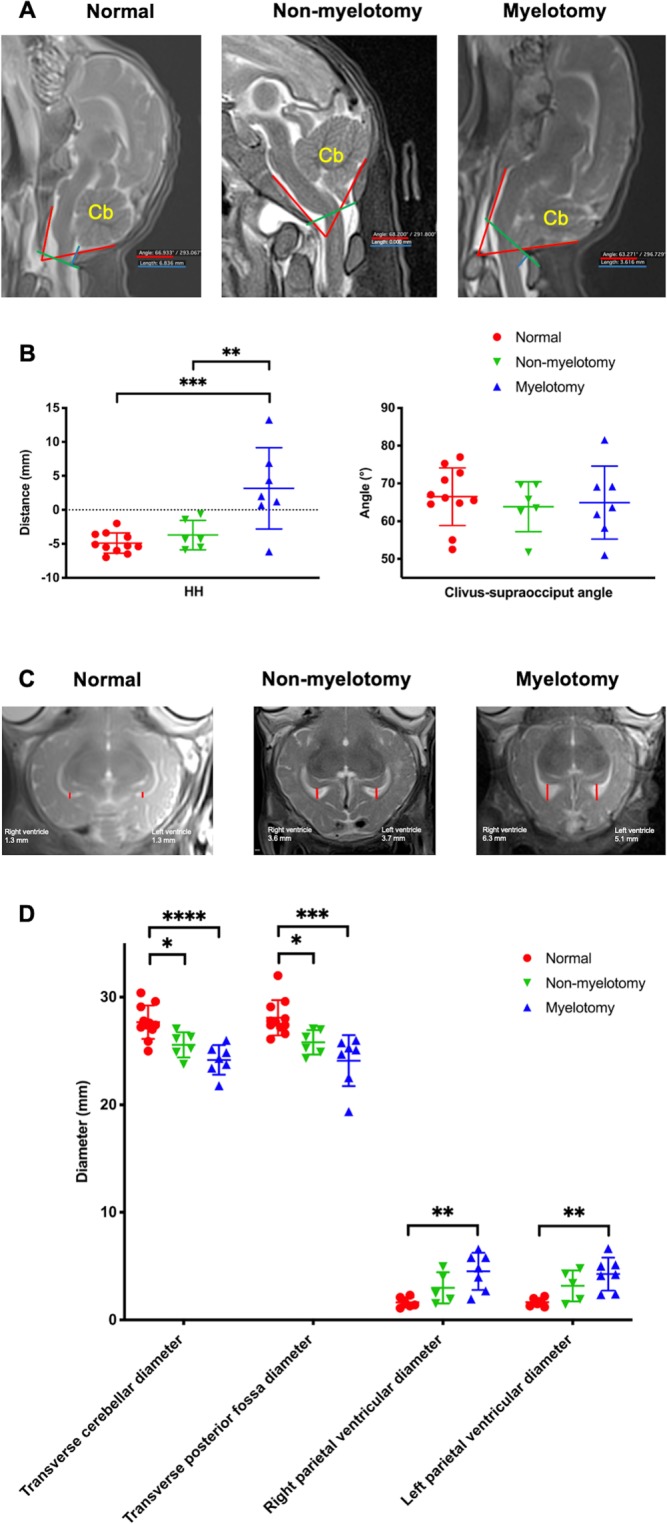
Magnetic Resonance Imaging (MRI) of the brain. (**A**) Measurements of the hindbrain herniation (HH) distance (blue lines), i.e. distance from tip of cerebellum (Cb) to foramen magnum (green lines), and the clivus-supraocciput angle (red lines) on mid-sagittal slices; (**B**) results of these outcome measures per group. On transverse slices, measurements of the transverse cerebellar diameter, transverse posterior fossa diameter and diameters of the right and left ventricles (red lines; **C**); (**D**) results of these outcome measures per group. [Significance as *0.05 ≥ p > 0.01; **0.01 ≥ p > 0.001; ***0.001 ≥ p > 0.0001; ****p ≤ 0.0001].

Relevant parameters (Table S2) were measured on OsiriX Lite software version 9.0 (Pixmeo SARL, Bernex, Switzerland) by two board-certified radiologists^41^. For the brain these encompassed: presence and distance in millimeters (mm) of hindbrain herniation defined as the descent of the tip of the cerebellar vermis below the level of the foramen magnum (line between the basion and opisthion) outside the skull on mid-sagittal slices (Fig. 3A)^42,43^; clivus-supraocciput angle on mid-sagittal slices as defined by D’Addario *et al*.^44^; transverse cerebellar diameter and transverse posterior fossa diameter in mm on transverse slices^45^; frontal, parietal and temporal ventricular diameter in mm of the right and left ventricles separately, in the axial plane on an axis perpendicular to that of the ventricle for the frontal and temporal horns, and in the true anteroposterior plane for the parietal horns (Fig. 3C)^45^; and the presence or absence of brain parenchymal ischemia and hemorrhage. For the spinal cord, these included tissue thickness in mm covering the defect where coverage was thinnest; presence of CSF between spinal cord and overlying tissue of SBA to assess tethering; and kyphosis angle in degree at apex of normal angulation, usually at lumbar level L3–4. For the urinary tract, measurements included bladder wall thickness at the bladder neck^46,47^ and grading of hydronephrosis based on a clinical neonatal hydronephrosis grading system^48^: grade 1, no fluid visible or fluid visible in renal pelvis; grade 2, distended renal pelvis and visible calyces; grade 3, distended renal pelvis and calyces; grade 4, distended ureters, renal pelvis and calyces. The upper hindlimb muscle area was measured on reconstructed axial slices at mid-distance between the hip and knee joints perpendicular on the long axis of the bone. In total, 20 parameters were measured (Table S2).

#### MEP recording

**Figure 4:**
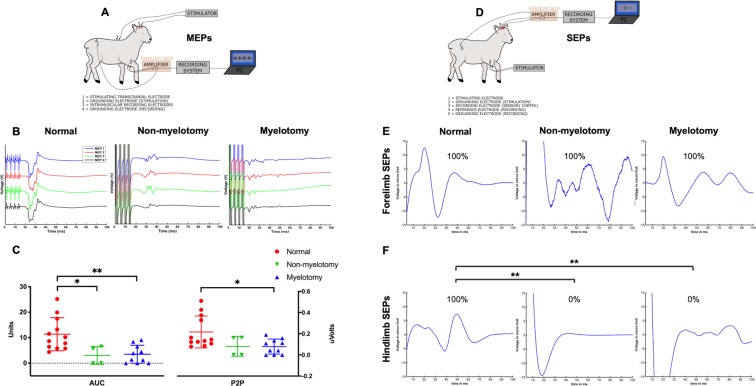
Motor Evoked Potentials (MEPs) and Somatosensory Evoked Potentials (SEPs) recordings and analysis. (**A**) Schematic illustration of the MEP experimental setup. The motor cortex was stimulated and the MEPs recorded in the distal contralateral limb muscles. (**B**) Visualization with a custom-made MATLAB MEP algorithm for each group. (**C**) Quantitative MEP analysis displaying the area-under-the-curve (AUC) and peak-to-peak amplitude (P2P). (**D**) Schematic illustration of the SEP experimental setup. The distal sensory nerve was stimulated and the SEPs recorded in the contralateral sensory cortex. Visualization with a custom-made MATLAB SEP algorithm of forelimb (**E**) and hindlimb (**F**) SEPs for each group. [Significance as *0.05 ≥ p > 0.01; **0.01 ≥ p > 0.001; ***0.001 ≥ p > 0.0001; ****p ≤ 0.0001]. Drawings (**A**,**D**) by M. Deprez for and copyright by UZ Leuven, Belgium.

Our MEP protocol has been described previously^38^. Briefly, MEPs were recorded between two needle electrodes inserted in the distal forelimb and hindlimb muscles following a contralateral motor cortex transcranial stimulation using skull screw electrodes (Fig. 4A). A standardized analysis method using a custom-made algorithm in MATLAB (Mathworks, Natick, MA, USA) was applied on the MEP raw data. Data were filtered between 30 and 1500 Hz using a second order Butterworth filter. The timing of stimulation onset was detected using the data from the trigger channel. A 100 ms time window was chosen and defined as the time between first pulse of the stimulus and 100 ms afterwards^49^. Using our designed Graphical User Interface, we extracted three reliable quantitative parameters, i.e. latency, area-under-the-curve (AUC) and peak-to-peak amplitude (P2P).

#### SEP recording

This consisted of a set-up similar to our MEP set-up yet reversing the simulation and recording areas and adapting the stimulus (Fig. 4D). Needle electrodes (22G x 2″, BD Microlance 3; Becton, Dickinson and Company (BD); Franklin Lakes, NJ, United States) were inserted percutaneously 1 cm apart along the ulnar or posterior tibial nerve to stimulate the somatosensory tracts. To record SEPs, two skull-screw electrodes (4.7 mm length–1.17 mm shaft diameter, #19010-00, Fine Science Tools (FST), North Vancouver, BC Canada) were bilaterally screwed over the somatosensory cortex. Two reference skull-screw electrodes were bilaterally screwed over the motor cortex. To prevent noise, the preamplifier was first wrapped in aluminum foil and then encased in a stainless steel ‘Faraday’ box which was connected to the lamb using a subcutaneous needle (18G x 1½″, BD Microlance 3) in the lumbar-sacral region.

We defined a stimulus as a unique bipolar and anodal pulse (pulse width of 0.1 ms, frequency of 3 Hz) that triggered one response, being an evoked potential. Electrical stimulation was applied between the two needle electrodes inserted in the distal ulnar or posterior tibial nerve and were spaced 1–2 cm apart from each other^50^. It was recorded contra-laterally between two skull-screw electrodes located above the somatosensory cortex. For each recording, a SEP was the average of 1,500 evoked potentials. MC-Rack software (recording system from Multi Channel Systems (MCS), Reutlingen, Germany) was used to average and visualize the SEP data. A 100 ms time window was chosen to display the SEP recordings. Two independent assessors blinded to the treatment groups analyzed the SEP data and defined in consensus whether contralateral hindlimb SEPs were present or absent.

#### Histology and immunohistochemistry

Lambs were euthanized and bled out via the jugular vein. After gross inspection, the SBA defect was resected *en-bloc* from T10 till the tail and the brain within the skull and neck down to C6. We also harvested the rectum and bladder of the lambs. They were opened them on the antimesenteric side, laid flat on histopathology paper (Macherey-Nagel & Co., Düren, Germany) and fixed. Also, a biopsy from both right and left tensor fasciae latae muscles, 2 cm away from the hip joint, was taken. Fixation was by immersion in 4% neutral phosphate buffered formaldehyde (Klinipath, Duiven, The Netherlands) for at least three months to dehydrate it completely^51^. Subsequently, the brain and brainstem were dissected out of the skull. The SBA defect was divided into six blocks, one per lumbar level. Alternatively, one level was assigned to assessment of spinal cord; the next one was assigned to evaluate the relationship between the cord and the overlaying layers. For spinal assessment (L1, L3, L5), the spinal cord was dissected out. For assessment of the overlaying tissues (L2, L4, L6) the blocks were decalcified for 10 hours with hydrochloric acid (Surgipath Decalcifier II, Leica Biosystems, Wetzlar, Germany). Specimens were embedded in paraffin and 7 µm sections were cut^42^ and prepared for hematoxylin and eosin (H&E) staining.

**Figure 5:**
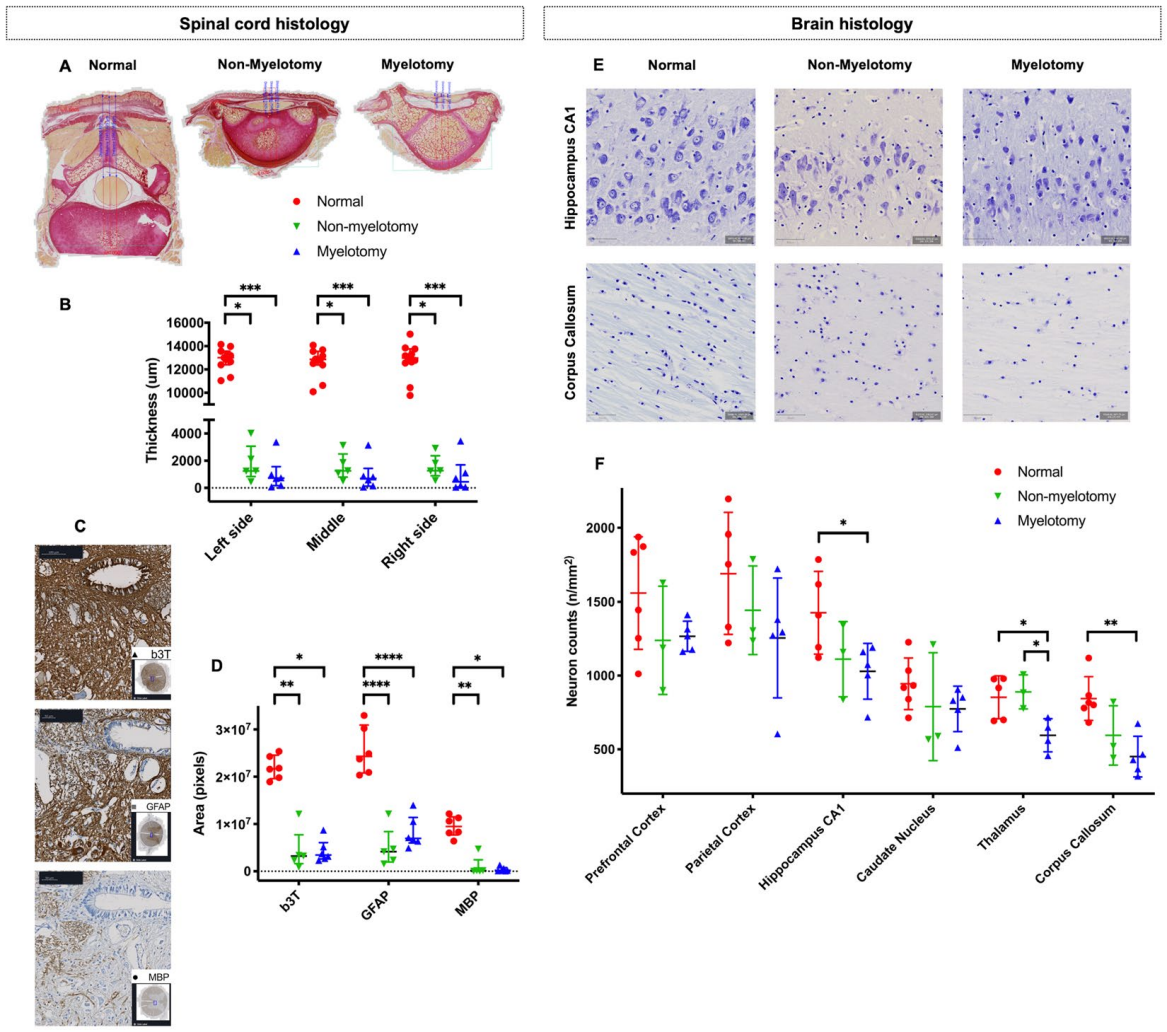
Histology and immunohistochemistry of the spinal cord and brain. (**A**) Spinal cord histology for each group, with thickness measurements of the tissue covering the spinal cord on the left, middle and right side (blue lines). (**B**) Graph of spinal cord thickness measurements. (**C**) Immunochemistry of the spinal cord for each group, with quantification of the area of the b3T, GFAP and MBP staining. (**D**) Graph of immunohistochemistry measurements. (**E**) Representative fields from brain slides stained with Cresyl violet (scale bar 50 µm). (**F**) Neuron densities per region of interest of the brain comparing the two surgical groups to the normal lambs. [Data displayed as mean and SD with significance as *0.05 ≥ p > 0.01; **0.01 ≥ p > 0.001; ***0.001 ≥ p > 0.0001; ****p ≤ 0.0001].

For the spinal cord, Sirius red stains were made on the blocks with overlaying tissues (L2 and 4 blocks surrounding the lesion epicenter^35^) to define the collagen layer covering the spinal cord and measure the distance between the spinal cord and the tissue cover on the right, middle and left (in-between distance of 1000 µm) of a transverse section (Fig. 5A). Immunohistochemistry was done on L3 blocks to evaluate the vitality of the spinal cord at its epicenter^35^. Immunohistochemistry was also performed. Anti-glial fibrillary acidic protein antibody (GFAP antibody Z0334, 1/4000, Dako, Agilent Technologies, Santa Clara, CA, United States) binds with mature glial cells such as astrocytes and some groups of ependymal cells in the gray and white matter. Expression of GFAP is tightly associated with astrocytic cell proliferation and differentiation^52,53^. In SBA, GFAP is considered as a marker for ongoing destruction^13^. Anti-neuronal beta-III tubulin antibody (b3T antibody G7121, 1/100, Promega Corporation, Madison, WI, USA) labels neurons anywhere in the central nervous system. As neurotubulin is one of the earliest markers of neurogenesis^54^, it was used to evaluate the degree of neural differentiation. Anti-Myelin Basic Protein antibody (MBP antibody sc-271524, 1/500, Santa Cruz Biotechnology, Dallas, TX, United States) labels myelin which is the most abundant protein in the myelin membrane. All sections were costained with DAB (3,3′-Diaminobenzidine) to visualize the nuclei as well as secondary antibodies combined with streptavidin-HRP (horseradish peroxidase) in the Avidin Biotin Complex method to block endogenous biotin and avoid background staining (HRP/DAP detection kit #ab64264; Abcam, Cambridge, UK). Each section was also stained without primary antibody as a control staining. Digital images were taken for analysis with the Zen lite software (version 2.3 for Windows, Carl Zeiss Microscopy, Jena, Germany) on Sirius red slides to measure the thickness of the collagen layer covering the spinal cord (Fig. 5A), and on H&E stains, the trigonal muscle 5 mm above the bladder neck, and of the circular and longitudinal layers around the posterior aspect of the rectal ampulla 5 mm above the upper border of the external anal sphincter, for both in the midline. We also assessed the tensor fasciae lata muscles to qualitatively categorize them on H&E stain as “normal” (absence of atrophy or hypertrophy or incidental (≤25%) muscle fiber hypertrophy) or “abnormal” (pronounced (>25%) muscle fiber atrophy and hypertrophy or muscle atrophy and interspersed fat and collagen deposition, i.e. fibrosis)^55^. For the spinal cord, we first converted czi files into tiff files using CZItoTIFFBatchConverter freeware (www.med.uio.no) and then converted and resized (40%) them into jpeg files using IrfanView freeware (www.irfanview.com). Areas (pixels) of positive b3T, GFAP and MBP immunostaining were measured automatically using Fiji-ImageJ software (version 2.0.0-rc-65/1.51u)^56^. Background was subtracted using automated color segmentation and thresholding of brightfield image (Fig. 5C)^57,58^.

Each brain was block dissected into 4 parts at about 1–2 cm intervals, creating a frontal (comprising of the prefrontal and frontal cortex), middle (comprising of the temporal-parietal cortex), occipital (comprising of the occipital cortex and midbrain) and cerebellar block. Blocks were serially sectioned at 4 µm slices at 200 µm intervals to evaluate the following 6 regions: the prefrontal cortex, parietal cortex, caudate nucleus, hippocampus, thalamus and corpus callosum (Fig. 5E)^59,60^. Neuron density quantification was done by Nissl staining (Cresyl Violet acetate C5042-10G, Sigma-Aldrich, St. Louis, MO, United States). From blocks 1 to 3 a set of 3–5 coronal sections were taken resulting in about 10–20 sections per animal. Histological slides were digitized using the Zeiss AxioScan Z1 imaging platform (AxioScan Slide Scanner, Carl Zeiss Microscopy; Fig. 5E). All focusing and field of view assemblies were handled by the Zen lite software, which is integrated with the AxioScan device. Quantification profiles on the digitized whole-slide images were done under bright field setting using QuPath freeware (qupath.github.io)^61^ and utilizing the fast cell counting. Furthermore, the detection classifier function was used with nucleus detection settings to differentiate between cellular types in each stain^62^.

In summary, 17 histological parameters or regions of interest from the spinal cord (n = 7), brain (n = 6), hindlimb muscles (n = 1), bladder (n = 1) and rectum (n = 2) were respectively measured or quantified (Table S2).

### Statistical methods

Data were collected in an Excel database file (Excel 15.28 for MacOs X, Microsoft Corporation, Redmond, WA, USA). Data processing and analysis was done twice for each group of outcome measures by two independent observers blinded to origin of the recording and the allocated experimental condition.

#### Reliability analysis

For all continuous data, we analyzed inter- and intra-rater reliability using the intraclass correlation coefficient (ICC) and the reliability coefficient Cronbach’s α (Table S2)^63,64^. Statistical analysis was performed with SPSS Statistics software version 21.0 for MacOs X (IBM Corporation, Amonk, NY, USA). We selected “absolute agreement” type and “two-way mixed” model. Finally, we used results from the “single measures” option because non-averaged values from two raters were always collected. Values of α ≥ 0.7^65^ and ICC ≥0.4^66,67^ were considered a reliable and clinically acceptable consensus. For categorical data, we measured intra-rater reliability with McNemar statistic and inter-rater reliability with Cohen’s kappa coefficient on SPSS^64,68^. A score ≥0.4 was considered a reliable and clinically acceptable consensus^69^.

#### Correlation analysis

We used GraphPad Prism version 7.00 for MacOs X (GraphPad, La Jolla, CA, USA) on outcome measurements that were reliably measured. The purpose was to determine the correlation between motor function outcome measurements of both non-myelotomy and myelotomy groups, i.e. joint movement score, the locomotor grade, the MEPs area-under-the-curve (AUC) and peak-to-peak (P2P), the area of spinal cord beta-III tubulin on immunohistochemistry as well as hindbrain herniation distance on MRI (Table S4). Pearson or Spearman correlation were used when the data of each non-myelotomy and myelotomy group had normal or abnormal distribution respectively (Table 1). A value of r ≥ 0.5 and ≥0.7 were considered moderate and strong correlations, i.e. moderate or strong uphill (positive) linear relationship^70^. When the two-tail P value was <0.05, we rejected the null hypothesis that the correlation was due to random sampling and the results were therefore significant.

**Table 1:** Comparison of all reliable outcome measures used to determine a spina bifida phenotype.

Groups	Normal	Non-myelotomy	P value Non-myel. vs. Normal	Myelotomy	P value Myel. vs. Normal	P value Myel. vs. Non-myel.
Survival at birth of open defects	71.4% (25/35)	54.5% (6/11)	0.501	70.0% (7/10)	0.758	0.781
**BRAIN PHENOTYPE**						
**Neurological examination**	**N** = **14**	**N** = **6**		**N** = **7**		
Abnormal spontaneous head movement (tremor/spasm)	7% (1/14)	83.3% (5/6)	**0**.**004**	85.7% (6/7)	**0**.**002**	0.514
**MRI**	**N** = **11**	**N** = **6**		**N** = **7**		
Presence of HH	0.0% (0/11)	0.0% (0/6)	1.000	85.7% (6/7)	**0**.**001**	**0**.**011**
HH distance (mm)	−4.9 ± 1.5	−3.7 ± 2.2	0.787	3.2 ± 6.0	**<0**.**001**	**0**.**006**
Clivus-supraocciput angle (°)	66.5 ± 7.6	63.8 ± 6.6	0.793	64.9 ± 9.7	0.914	0.968
Transverse cerebellum diameter (mm)	27.7 ± 1.6	25.6 ± 1.2	**0**.**022**	24.2 ± 1.4	**<0**.**001**	0.204
Transverse posterior fossa diameter (mm)	27.7 (1.9)	25.8 (2.2)	**0**.**033**	25.1 (3.3)	**<0**.**001**	1.000
Right Parietal ventricle diameter (mm)	1.6 ± 0.5	3.0 ± 1.5	0.255	4.5 ± 1.7	**0**.**004**	0.162
Left Parietal ventricle diameter (mm)	1.7 ± 0.4	3.2 ± 1.5	0.143	4.3 ± 1.5	**0**.**005**	0.314
Presence of brain hemorrhage	0% (0/11)	0% (0/6)	1.000	0% (0/7)	1.000	1.000
Presence of brain ischemia	0% (0/11)	0% (0/6)	1.000	14% (1/7)	0.815	0.936
**Histology**	**N** = **6**	**N** = **3**		**N** = **5**		
Density of prefrontal cortex (neurons/mm^2^)	1560 ± 381	1240 ± 367	0.339	1268 ± 103	0.298	0.991
Density of parietal cortex (neurons/mm^2^)	1692 ± 412	1443 ± 300	0.668	1256 ± 407	0.229	0.792
Density of hippocampus (neurons/mm^2^)	1427 ± 280	1112 ± 255	0.184	1030 ± 189	**0**.**049**	0.889
Density of caudate nucleus (neurons/mm^2^)	945 ± 175	790 ± 366	0.585	774 ± 155	0.425	0.995
Density of thalamus (neurons/mm^2^)	853 ± 145	890 ± 116	0.922	595 ± 113	**0**.**037**	**0**.**036**
Density of corpus callosum (neurons/mm^2^)	844 ± 148	594 ± 201	0.102	451 ± 137	**0**.**004**	0.443
**SPINAL CORD PHENOTYPE**						
**Gross examination**	**N** = **14**	**N** = **6**		**N** = **7**		
Size of defect (mm)						
- Length	0.0 (0.0)	22.0 (22.3)	**0**.**012**	77.0 (20.0)	<**0**.**001**	0.453
- Width	0.0 (0.0)	11.0 (12.5)	**0**.**008**	30.0 (40.0)	<**0**.**001**	0.680
Lumbar CSF leakage	0/14 (0%)	3/6 (50%)	**0**.**029**	7/7 (100%)	**<0**.**001**	0.141
**MRI**	**N** = **11**	**N** = **6**		**N** = **7**		
Kyphosis angle (°)	169 ± 8	142 ± 22	**0**.**005**	147 ± 11	**0**.**011**	0.794
**Histology**	**N** = **6**	**N** = **5**		**N** = **6**		
SC adhesions	0.0% (0/11)	80.0% (4/5)	**0**.**005**	83.3% (5/6)	**0**.**002**	0.521
Coverage thickness (µm)						
Left	13010 (1176)	1248 (2224)	**0**.**020**	726 (1412)	**<0**.**001**	1.000
Middle	12846 (1173)	1257 (1696)	**0**.**020**	706 (1308)	**<0**.**001**	1.000
Right	12975 (1212)	1275 (1467)	**0**.**022**	448 (1629)	**<0**.**001**	1.000
Neuronal cells (b3t) area (10^6^ pixels)	21.7 (4.9)	3.2 (6.1)	**0**.**011**	3.4 (3.5)	**0**.**015**	1.000
Negative control	1.7 (2) × 10^3^					
Astroglial cells (GFAP) area (10^6^ pixels)	25.5 ± 5.1	5.0 ± 4.2	**<0**.**001**	8.3 ± 3.3	**<0**.**001**	0.431
Negative control	4.2 ± 4 × 10^3^					
Myelin (MBP) area (10^6^ pixels)	9.5 (3.8)	0.1 (2.4)	**0**.**006**	0.2 (0.7)	**0**.**026**	1.000
Negative control	1.7 (2) × 10^3^					
**HINDLIMB PHENOTYPE**						
**Neurological examination**	**N** = **14**	**N** = **6**		**N** = **7**		
Abnormal gait (paraparesis or paraplegia)	0% (0/12)	100% (6/6)	**<0**.**001**	100% (7/7)	**<0**.**001**	1.000
Lumbar-sacral sensory deficit	0% (0/14)	100% (6/6)	**<0**.**001**	100% (7/7)	**<0**.**001**	1.000
**HL neuromotor deficit**	**N** = **13**	**N** = **5**		**N** = **7**		
- Joint movement score (/12)	12.0 ± 0	7.2 ± 5.35	**0**.**004**	3.2 ± 2.19	**<0**.**001**	**0**.**039**
- Locomotor grade (/15)	14.2 ± 0.34	3.0 ± 2.26	**<0**.**001**	1.4 ± 0.72	**<0**.**001**	**0**.**044**
**Hindlimb MEP**	**N** = **6** × **2**	**N** = **2** × **2**		**N** = **5** × **2**		
Threshold (Volts)	25 (15)	80 (51)	NA	45 (41)	NA	
AUC	11.4 ± 6.48	3.0 ± 3.53	**0**.**027**	3.5 ± 3.54	**0**.**005**	0.987
P2P (uVolts)	0.22 ± 0.15	0.08 ± 0.09	0.113	0.08 ± 0.07	**0**.**036**	0.981
**Hindlimb SEP**	**N** = **6** × **2**	**N** = **3** × **2**		**N** = **2** × **2**		
Forelimbs	100% (6/6)	100% (3/3)	1.000	100% (2/2)	1.000	1.000
Hindlimbs	100% (6/6)	0% (0/3)	**0**.**003**	0% (0/2)	**0**.**005**	1.000
**Histology**	**N** = **6** × **2**	**N** = **4** × **2**		**N** = **4** × **2**		
Proximal bilateral HL muscle atrophy	0% (0/6)	25% (1/4)	0.830	50% (2/4)	0.259	1.000
**BLADDER AND RECTUM PHENOTYPE**						
**Clinical examination**	**N** = **14**	**N** = **7**		**N** = **7**		
Urinary incontinence	7.1% (1/14)	83.3% (5/6)	**0**.**004**	85.7% (6/7)	**0**.**002**	0.514
**Histological examination**	**N** = **2**	**N** = **1**		**N** = **1**		
Thickness of bladder muscle (µm)	841 ± 293	1209	NA	1298	NA	NA
Thickness of rectum muscle (µm)						
- Circular layer	500 ± 180	558	NA	860	NA	NA
- Longitudinal layer	377 ± 197	368	NA	696	NA	NA

Binomial and ordinal variables were expressed as percentage and score. Continuous variables normally distributed were expressed as mean ± standard deviation (SD) and those not normally distributed were expressed as median and interquartile range (IQR). Some lambs were too weak to get more results than from the neurological examination and MRI. Abbreviations: Myel., myelotomy; Non-myel. Non-myelotomy; HH, hindbrain herniation; SC, spinal cord; HL, hindlimb; FL, forelimb; MEP, motor evoked potentials; AUC, area-under-the-curve; P2P, peak-to-peak; SEP, somatosensory evoked potentials; b3t, beta-III tubulin antibody; GFAP, glial fibrillary acidic protein antibody antibody; MBP, myelin binding protein antibody; NA, non-applicable; NS, non-specified. Significant results are highlighted in bold.

#### Group comparison

GraphPad Prism was also used on reliable outcome measurements. Binomial and ordinal variables were expressed as percentage and score, respectively. Chi-square test with Yates’ correction was used to compare them. Continuous variables were tested for normal distribution using the D’Agostino-Pearson (omnibus K2) normality test^71^. We assumed that all our measurements showed equality of variance as the groups had the same sample size^72,73^. Those normally distributed were presented as mean and standard deviation (SD) and compared with one-way analysis of variance (ANOVA) combined with post hoc Dunnett’s multiple comparison test. Continuous variables not normally distributed were expressed as median and interquartile range (IQR) and compared with the Kruskal-Wallis test combined with post hoc Dunn’s multiple comparison test. A p value <0.05 was considered significant.

## Results

Our study included a total of 33 ewes and 57 fetuses (6 triplets, 11 twins and 17 singletons) from three groups. Twelve singletons were recruited as controls, together with 23 fetuses from multiplets (total: 35 controls in the normal group). There were 12 fetuses in the non-myelotomy and 10 in the myelotomy group. Survival at birth (pooled mean of 67%) was comparable in the three groups (Table 1).

### Reliability analysis of outcome measures

From the 38 neurological examination parameters assessed, 6 objective and discriminant ones were withheld (Table S2). Ten out of 20 magnetic resonance imaging (MRI) variables were reliable. For the MEPs, two out of three variables were reliable (AUC and P2P). For the SEPs and histology, all were reliable.

### Correlation analysis of motor function outcomes

For the myelotomy group, a strong correlation (r > 0.85; p < 0.05) was demonstrated between join movement score, locomotor grade and P2P of the MEPs (Table S4). A trend towards significant correlation was observed between these outcomes and AUC of the MEPs (r = 0.86; p = 0.06). In contrast, a strong correlation (r = 1; p < 0.05) was seen for the non-myelotomy group between join movement score and locomotor grade. There was however no correlation between these outcomes and b3T area on immunohistochemistry as well as hindbrain herniation.

### Phenotype of the model with myelotomy

All lambs were included as none had a complete spontaneous skin coverage of the defect at birth (Fig. 2A,B and Table 1). The lambs presented with the typical brain phenotype including hindbrain herniation, ventriculomegaly, smaller than normal posterior fossa and cerebellum and absence of subacute or chronic hemorrhage or ischemia on MRI (Fig. 3 and Table 1). Histology of the brain confirmed the absence of parenchymal hemorrhage or ischemia and showed a decreased number of neurons in the hippocampus, thalamus and corpus callosum (Fig. 5F). They also had the spinal cord phenotype consisting of a large (median of 77 × 30 mm; IQR of 20 × 40) open lumbar defect with CSF leakage (Fig. 2A,B) partially covered with a thin layer of tissue fibrosis, kyphosis on MRI and extensively damaged spinal cord with loss of neuronal, and astroglial cells and myelin on histology (Fig. 5A–D). Their hindlimb phenotype was a sensory-motor deficit with consistent paraparesis or paraplegia (Fig. 2C,D) confirmed by the absence of MEPs (Fig. 4B,C) and SEPs (Fig. 4E,F) and pronounced muscle atrophy and hypertrophy on histology. Clinically, the lambs presented with urinary incontinence. No formal conclusions on the histologic bladder and rectal phenotype could be drawn because of lack of power (Table S2). Additionally, there was homogeneity between the lambs as the variability (SD or IQR) of the outcomes was low (Table 1). Overall, the myelotomy group simulated comprehensively the pathophysiology (second hit), anatomy and symptomatology of SBA, obtaining a validity score of 17/20 (Table S1). In conclusion, surgical induction of SBA including myelotomy resulted in a complete and homogeneous phenotype of a non-cystic lumbar L1–L6 SBA.

### Phenotype of the model without myelotomy

One lamb out of 7 was excluded from final analysis due to complete spontaneous skin coverage of the defect at birth (Fig. 2A,B and Table 1). In the remaining six lambs without myelotomy, the complete pathophysiology and symptomatology of SBA was not reproduced (validity score of 15/20; Table S1). They did not consistently have hindbrain herniation and ventriculomegaly, posterior fossa and cerebellum anomalies on MRI (Fig. 3) nor did they have complete clinical hindlimb motor and sensory deficit (Fig. 2C,D) confirmed by the inconsistent absence of MEPs (Fig. 4B,C) and SEPs (Fig. 4E,F) and inconsistent abnormal muscular trophicity on histology (Table 1). The lambs also displayed urinary incontinence (Table 1). Induction of SBA without myelotomy resulted in smaller but variable open lumbar defects (median of 22 × 11 mm; IQR of 22 × 13) with CSF leakage (Fig. 2A,B), variable posterior fossa abnormalities yet consistently without subacute or chronic hemorrhage or ischemia and kyphosis on MRI (Fig. 3). On histology, despite an extensively damaged spinal cord completely covered with a thick layer of tissue fibrosis (Fig. 5A–D), there was neither indication of brain hemorrhage or ischemia, nor decreased number of brain neurons (Fig. 5F). Overall, there was heterogeneity between the lambs as the variability of the outcomes was high (Table 1). In summary, induction of SBA without myelotomy displayed a partial and heterogeneous phenotype of a lumbar L1–L6 SBA, i.e. a heterogeneous *in utero* lumbar spinal cord injury.

## Discussion

### Main findings

An appropriate animal model should mimic the human condition in its etiology, pathophysiology, symptomatology and response to therapeutic interventions^24–26^. In this study, we introduced a standardized method to comprehensively and reliably characterize the spina bifida phenotype at birth in the two existing lamb surgical models. Using that method, we showed that the myelotomy model best phenocopied the entire pathophysiology, anatomy and symptomatology of non-cystic lumbar SBA. Because previous experiments have shown that a two-layer fetal repair reverses this sequence of events^11^, it obtains the highest validity score for animal models of SBA (17/20; Table S1)^23^. The non-myelotomy model does not mimic a SBA but a heterogeneous *in utero* lumbar spinal cord injury.

### Meaning of the study

Since the 1980’s, numerous SBA preclinical disease models have been developed. Small ones such as rodents and rabbits^32,34,74–76^ were meant to improve our understanding of the biological mechanisms and pathophysiology, screening new covering implant materials for biocompatibility and evaluating fetal therapies^27,77,78^. Despite their high validity (scores of 16/20 (mouse), 16/20 (rat), 14/20 (rabbit) respectively; Table S1), they are not suitable to evaluate feasibility, safety and efficacy of any fetal therapy due to their small size and the different physiology. In an attempt to translate observations made in these small animal models, large models are required. Those include non-human primates (rhesus monkey)^79^ and sheep, in which a large, multilayer defects is induced, with or without myelotomy^10,11,16^. Another sheep model, the full-thickness skin defect fetal lamb model (score of 10/20), have been also developed specifically to test the feasibility of new fetal techniques^80,81^. If no myelotomy is performed at the time of induction of SBA these models obtain a lower validity score than the rat (15/20 vs. 16/20; Table S1) yet also than the myelotomy sheep model (17/20). This does not mean that these other models have no value. For instance, in the non-myelotomy sheep model, the induction causes a variable yet wide range of somatosensory effects which may partly mimic the SBA clinical presentation.

### Strengths and limitations

Our study first followed the international guidelines for animal research and for validation of animal disease models^24–26^. Secondly, the experiment was well powered and all the outcome measures used were obtained by a multidisciplinary research team with observers as much as possible blinded to the treatment groups, and only those outcomes shown to be reliably measurable, were used in the analysis. This will make implementation in other hands and experiments more reproducible. Thirdly, by inducing an important spinal CSF leakage, this myelotomy model displays the macroscopic and microscopic brain SBA phenotype, encompassing a Chiari II malformation (triad of hindbrain herniation, small posterior fossa and small cerebellum), ventriculomegaly, abnormalities of corpus callosum, hippocampus and thalamus^21,59^. This confirms the unified theory explaining the pathogenesis of the Chiari II malformation and the posterior fossa anomalies in SBA^15–17^. Fourthly, the model phenocopies a non-cystic SBA which is clinically referred to as myeloschisis as opposed to myelomeningocele and occurs in about one third of the SBA children^82^. Finally, we demonstrated the homogeneity of this model unlike the one without myelotomy, confirming earlier observations by Brown *et al*.^83^. This is of high relevance to design and power animal experiments in this field.

We are also aware of limitations to our study. Firstly, surgical inductions were not identical in the two groups. In the model without myelotomy, the skin resection was oval (≥3 × 4 cm) compared to the circular resection (≥4 × 4 cm) in the myelotomy model. This was because we followed the surgical protocol of most of the other research teams worldwide^23^. Without myelotomy, the skin has the potential to spontaneously heal in utero. Therefore, and to lower the risk of selection bias, we excluded from the analysis observations of one lamb that had a complete skin closure at birth. Secondly, diffusion tensor imaging (DTI) and susceptibility-weighted MRI sequences to detect subtle and acute hemorrhagic and ischemic brain changes could not be accomplished due to problems in optimization the setup thereof. Nonetheless, the latter changes were not identified on histological examination and no major subacute and chronic changes were detected on anatomical T2- and T1-weighted sequences. Future research will include DTI as a bioimaging marker to better evaluate the microstructural alterations in the brain^84^. Thirdly, not all lambs did survive until the MEPs and SEPs recording, potentially introducing selection bias. Nevertheless, the MEPs had a high correlation with the neurological examination. This could be explained by the fact that the lambs assessed were the ones with the worst joint scores and motor grades. Fourthly, histopathological protocols used in this research underwent a learning curve with the result that the initial specimens could not be included due to poor fixation and preparations protocols. Yet the analysis method was shown reliable on the pooled results of the herein reported animals, and can therefore be used in further studies. Fifthly, this myelotomy model does not allow to assess the somatosensory effects on the spinal cord because myelotomy per definition disrupts the posterior somatosensory tracts^23^. It is therefore most useful to evaluate the impact on the brain and spinal cord motor function. Conversely, the non-myelotomy model, despite its heterogeneity, is more adequate for examination of distal spinal cord function^35^. Sixthly, the brain and spinal cord sequelae following myelotomy might limit the utility of this model for long-term studies. Following the fetal repair, the lambs remain handicapped and are difficult to nurse. Seventhly, the non-human primate remains the gold standard model for uterine and fetal manipulation. Nonetheless, a SBA model has not yet been developed despite the existing primate model of *in utero* lumbar spinal cord injury (Table S1)^79^. The fetal lamb model has a totally different placentation, amniotic membranes which are not attached, and its repair at 100 days corresponds to 26 weeks of gestation in humans, the latest time point for fetal repair^10,39^. It therefore cannot be used to test the effect of early fetal interventions at around 16 weeks, which are currently also considered. Finally, this model is not adequate to assess preventative strategies due its surgical etiology which is not comparable to human SBA.

## Conclusion

Using a comprehensive and reliable standardized method, we demonstrated that the fetal lamb myelotomy model phenocopies a human non-cystic lumbar SBA. This model is at present the SBA animal model with the highest validity score. We also showed that the non-myelotomy model does not phenocopy a SBA yet an *in utero* spinal cord injury. In future studies, we propose to use the myelotomy model to test the efficacy of novel medical or surgical fetal interventions for SBA, such as fetoscopic techniques and tissue-engineered patches that can be seeded with stem cells or growth factors, and to train fetal surgeons for these procedures. When needed, the non-myelotomy model could be a surrogate model to evaluate the distal spinal cord function.

## Supplementary information

Supplementary information

## Data Availability

The datasets generated during and/or analyzed during the current study are available in the Supplementary Materials. The custom-made MATLAB algorithm is available upon request.
