# Supplementary material for: Validation of the Fetal Lamb Model of Spina Bifida
Source: Sci Rep. 2019 Jun 27;9:9327. doi: 10.1038/s41598-019-45819-3 (PMC6597719; doi:10.1038/s41598-019-45819-3)
Supplement: Supplementary file 1 — Supplementary information [file 41598_2019_45819_MOESM1_ESM.docx]

**Title**

Validation of the fetal lamb model of spina bifida

**Authors**

Luc Joyeux^1,2^, Alexander C. Engels^1-3^, Johannes Van Der Merwe^1,2,4^, Michael Aertsen^5^, Premal A. Patel^6^, Marjolijn Deprez^7^, Ahmad Khatoun^8^, Savitree Pranpanus^1,9^, Marina Gabriela Monteiro Carvalho Mori da Cunha^1^, Stephanie De Vleeschauwer^10^, Johanna Parra^11^, Katerina Apelt^1^, Myles Mc Laughlin^8^, Frank Van Calenbergh^12^, Enrico Radaelli^13^, Jan Deprest^1,2,4,14 *^

**Affiliations**

^1^Department of Development and Regeneration, Cluster Urogenital, Abdominal and Plastic surgery, Biomedical Sciences, Katholieke Universiteit (KU) Leuven, Leuven, Belgium

^2^Center for Surgical Technologies, Faculty of Medicine, KU Leuven, Leuven, Belgium

^3^Department of Obstetrics and Gynecology, University Hospital Bonn, Bonn, Germany

^4^Department of Obstetrics and Gynecology, Division Woman and Child, Fetal Medicine Unit, University Hospital Gasthuisberg, Leuven, Belgium

^5^Department of Radiology, University Hospital Gasthuisberg, Leuven, Belgium

^6^Radiology Department, Great Ormond Street Hospital for Children, NHS Foundation Trust, London, United Kingdom

^7^Research group Experimental Neurosurgery and Neuroanatomy, Department of Neurosciences, KU Leuven, Leuven, Belgium

^8^Experimental Otorhinolaryngology, Department of Neurosciences, KU Leuven, Leuven, Belgium

^9^Department of Obstetrics and Gynecology, Faculty of Medicine, Prince of Songkla University, Hat Yai, Songkhla, Thailand.

^10^Animal Research Center, Biomedical Sciences, KU Leuven, Leuven, Belgium

^11^Barcelona Center for Maternal Fetal and Neonatal Medicine, University of Barcelona, Barcelona, Spain

^12^Department of Neurosurgery, University Hospital Gasthuisberg, Leuven, Belgium

^13^Department of Pathobiology, Ryan Veterinary Hospital, University of Pennsylvania School of Veterinary Medicine, Philadelphia, PA, United States

^14^Institute of Women’s Health, University College London Hospitals, London, United Kingdom

**Corresponding author**

Jan Deprest, M.D. Ph.D.

Academic Department Development and Regeneration, Cluster Woman and Child,

Biomedical Sciences, Faculty of Medicine, Katholieke Universiteit KU Leuven

UZ Herestraat 49, box 1034, 3000 Leuven, Belgium

E-Mail : jan.deprest@uzleuven.be

**Supplementary information**

**Table S1- Scoring system developed to describe the degree of validity of a disease model using five parameters:** (1) species, (2) complexity, (3) disease simulation, (4) face validity and (5) predictivity. In the right columns are the scores of the spina bifida models. Each parameter gets a score of 1 to 4 leading to a total maximum score of 20. *Adapted from Denayer et al., 2014.*

| **Parameter** | **Definition** | **Value** | **Score** | **Mouse genetic model** | **Rat terato-genic model** | **Rabbit surgical model** | **Primate surgical model** | **Sheep model without myelotomy assessed with our method** | **Sheep model with myelotomy assessed with our method** |
| --- | --- | --- | --- | --- | --- | --- | --- | --- | --- |
| **Species** | Animal species used | Human  Non-human primate  Non-human mammal  Non-mammal | 4  3  2  1 | 2 | 2 | 2 | 3 | 2 | 2 |
| **Complexity** | Complexity of the animal model on the scale of life | In vivo  Tissue  Cellular  Sub-cellular/molecular | 4  3  2  1 | 4 | 4 | 4 | 4 | 4 | 4 |
| **Disease simulation** | Extent of simulation of the pathophysiology and anatomy of the disease | True  Complex  Pharmacological  /surgical  No | 4  3  2  1 | 4 | 3 | 2 | 2 | 2 | 3 |
| **Face validity** | Extent of simulation of the disease symptomatology defined in the ICD10-CM | >1 core symptom  1 core symptom  1 symptom  No | 4  3  2  1 | 3 | 4 | 3 | 3 | 3 | 4 |
| **Predictivity** | Degree of response to treatment (efficacy) | Graded response for all treatments  Graded response for certain treatments  All-or-none response for certain treatments  No response or not shown | 4  3  2  1 | 3 | 3 | 3 | 3 | 4 | 4 |
| **Overall score of each spina bifida model** | | | | **16/20** | **16/20** | **14/20** | **15/20** | **15/20** | **17/20** |

**Table S2 – Reliability analysis.** For categorical variables McNemar and Cohen’s Kappa coefficients were used. For continuous variables, we measured Cronbach’s α coefficient and intra-observer and inter-observer intraclass correlation coefficients with 95% confidence intervals.

*Abbreviations: SBA, spina bifida aperta; ICC, intra-class correlation; intra-ICC, intraobserver intraclass correlation coefficient; inter-ICC, interobserver intraclass correlation coefficient; CI, 95% confidence interval; α, reliability coefficient (Cronbach’s α); McN, McNemar statistic; k, Cohen’s kappa coefficient statistic; HL, hindlimb; FL, forelimb; MEP, motor evoked potentials; AUC, area-under-the-curve; P2P, peak-to-peak; SEP, somatosensory evoked potentials; NA, non-applicable; NS, non-specified.*

| **Groups** | **Normal** | | **Non-myelotomy SBA** | | **Myelotomy SBA** | |
| --- | --- | --- | --- | --- | --- | --- |
| **Variables** | **Intra-α or McN**  **Intra-ICC (CI) or p value** | **Inter-α or k**  **Inter-ICC (CI)** | **Intra-α or McN**  **Intra-ICC (CI) or p value** | **Inter-α or k**  **Inter-ICC (CI)** | **Intra-α or McN**  **Intra-ICC (CI) or p value** | **Inter-α or k**  **Inter-ICC (CI)** |
| **Gross examination** | N=14 |  | N=6 | | N=7 | |
| **Size of the defect** | NA | 1  NA | NA | 1  NA | NA | 1  NA |
| **Leakage of the lumbar defect** | NA | 1  NA | NA | 1  NA | NA | 1  NA |
| **Neurological examination** | N=14 |  | N=6 | | N=7 | |
| **Abnormal spontaneous head movement** | NA | 1  NA | NA | 1  NA | NA | 1  NA |
| **Abnormal gait** | 1  p=1.0 | 1  NA | 1  p=1.0 | 1  NA | 1  p=1.0 | 1  NA |
| **Urinary incontinence** | 1  p=1.0 | 1  NA | 1  p=1.0 | 1  NA | 1  p=1.0 | 1  NA |
| **Lumbar-sacral sensory deficit** | NA | 1  NA | NA | 1  NA | NA | 1  NA |
| **HL joint movement score** | 1.00  1.00 (NA) | 1.00  1.00 (NA) | 0.99  0.99 (0.95-1.00) | 0.96  0.92 (0.74-0.98) | 0.77  0.61 (0.17-0.85) | 0.89  0.80 (0.50-0.93) |
| **HL motor grade** | 0.83  0.70 (0.43-0.85) | 0.64  0.48 (0.12-0.73) | 1.00  1.00 (NA) | 0.98  0.96 (0.84-0.99) | 0.60  0.42 (-0.08-0.76) | 0.88  0.78 (0.45-0.92) |
| **MRI** | N=11 |  | N=6 | | N=7 | |
| **Presence of HH** | 1  p=1.0 | 1  NA | 1  p=1.0 | 1  NA | 1  p=1.0 | 1  NA |
| **Distance HH** | 0.95  0.90 (0.79-0.96) | 0.81  0.69 (0.39-0.86) | 0.96  0.94 (0.79-0.98) | 0.89  0.77 (0.39-0.93) | 1.00  1.00 (0.99-1.00) | 1.00  0.99 (0.98-1.00) |
| **Clivus-supraocciput angle** | 0.71  0.53 (0.16-0.77) | 0.76  0.61 (0.27-0.81) | 0.84  0.72 (0.29-0.91) | 0.56  0.36 (-0.16-0.75) | 0.97  0.91 (0.72-0.97) | 0.90  0.61 (-0.09-0.88) |
| **Transverse cerebellum diameter** | 0.84  0.72 (0.42-0.88) | 0.70  0.48 (0.07-0.76) | 0.93  0.83 (0.43-0.96) | 0.72  0.58 (-0.03-0.87) | 0.86  0.74 (0.37-0.91) | 0.77  0.52 (0.01-0.82) |
| **Transverse posterior fossa diameter** | 0.85  0.74 (0.46-0.89) | 0.77  0.58 (0.19-0.81) | 0.89  0.79 (0.41-0.94) | 0.82  0.68 (0.21-0.90) | 0.90  0.76 (0.36-0.92) | 0.83  0.72 (0.32-0.90) |
| **Right Frontal ventricle diameter** | 0.74  0.53 (0.03-0.82) | 0.79  0.44 (-0.11-0.80) | 0.75  0.63 (0.05-0.89) | 0.52  0.31 (-0.22-0.74) | 0.58  0.41 (-0.11-0.76) | -0.13  -0.06 (-0.54-0.46) |
| **Left Frontal ventricle diameter** | 0.89  0.80 (0.48-0.93) | 0.88  0.68 (0.09-0.91) | 0.92  0.86 (0.55-0.96) | 0.83  0.62 (0.03-0.88) | 0.10  0.05 (-0.44-0.54) | 0.19  0.11 (-0.47-0.60) |
| **Right Parietal ventricle diameter** | 0.86  0.76 (0.36-0.92) | 0.92  0.82 (0.44-0.95) | 0.97  0.94 (0.80-0.99) | 0.97  0.92 (0.70-0.98) | 0.97  0.94 (0.81-0.98) | 0.68  0.51 (0.02-0.81) |
| **Left Parietal ventricle diameter** | 0.85  0.75 (0.35-0.92) | 0.83  0.71 (0.28-0.91) | 0.94  0.88 (0.62-0.97) | 0.90  0.82 (0.45-0.95) | 0.95  0.91 (0.75-0.97) | 0.92  0.79 (0.31-0.94) |
| **Right Temporal ventricle diameter** | 0.87  0.76 (0.38-0.92) | 0.82  0.65 (0.15-0.88) | 0.84  0.70 (0.22-0.91) | 0.79  0.65 (0.11-0.89) | 0.82  0.65 (0.22-0.87) | 0.23  0.13 (-0.43-0.61) |
| **Left Temporal ventricle diameter** | 0.87  0.78 (0.39-0.93) | 0.89  0.81 (0.46-0.94) | 0.92  0.84 (0.49-0.96) | 0.59  0.40 (-0.21-0.80) | 0.58  0.41 (-0.13-0.76) | 0.76  0.62 (0.15-0.86) |
| **Presence of brain hemorrhage** | 1  p=1.0 | 1  NA | 1  p=1.0 | 1  NA | 1  p=1.0 | 1  NA |
| **Presence of brain ischemia** | 1  p=1.0 | 1  NA | 0.91  p=1.0 | 1  NA | 1  p=1.0 | 1 NA |
| **Tissue thickness covering defect where coverage is thinnest** | 0.87  0.77 (0.49-0.91) | 0.81  0.69 (0.33-0.87) | 0.86  0.77 (0.38-0.93) | -0.37  -0.12 (-0.48-0.40) | 0.97  0.94 (0.82-0.98) | 0.00  0.00 (-0.34-0.44) |
| **CSF presence between spinal cord & tissue coverage** | 1  p=1.0 | 1  NA | 1  p=1.0 | 1  NA | 0.93  p=1.0 | 0.06  NA |
| **Kyphosis angle** | 0.91  0.84 (0.63-0.94) | 0.90  0.79 (0.48-0.92) | 1.00  0.99 (0.95-1.00) | 0.99  0.97 (0.87-0.99) | 0.98  0.94 (0.79-0.98) | 0.94  0.87 (0.62-0.96) |
| **Right HL muscle area** | 0.97  0.95 (0.70-0.99) | -2.24  -0.02 (-0.03-0.12) | 0.99  0.97 (0.61-1.00) | 0.36  0.01 (-0.02-0.30) | 0.97  0.93 (0.79-0.98) | 0.76  0.19 (-0.07-0.59) |
| **Left HL muscle area** | 0.98  0.97 (0.82-1.00) | -0.12  -0.00 (-0.01-0.11) | 0.96  0.92 (0.42-0.99) | 0.74  0.02 (-0.01-0.35) | 0.98  0.96 (0.86-0.99) | 0.81  0.18 (-0.05-0.57) |
| **Bladder wall thickness** | 0.78  0.63 (0.30-0.83) | -0.26  -0.08 (-0.34-0.28) | 0.87  0.79 (0.30-0.95) | 0.53  0.12 (-0.10-0.52) | 0.69  0.53 (-0.03-0.84) | 0.15  0.03 (-0.11-0.32) |
| **Hydronephrosis grade** | 0.91  p=0.5 | 1  NA | 0.58  NA | 0.27  NA | 0.86  p=0.37 | 0.32  NA |
| **MEP** | **N=12** | | **N=2** | | **N=10** | |
| **Forelimb-MEP** |  |  |  |  |  |  |
| **Latency** | 0.91  0.83 (0.76-0.89) | 0.84  0.73 (0.62-0.81) | 0.81  0.68 (0.41-0.84) | 0.74  0.60 (0.29-0.79) | 0.98  0.96 (0.93-0.97) | 0.97  0.94 (0.90-0.96) |
| **AUC** | 1.00  0.99 (0.99-0.99) | 0.99  0.98 (0.97-0.99) | 1.00  1.00 (0.99-1.00) | 1.00  1.00 (0.98-1.00) | 0.99  0.98 (0.96-0.99) | 0.99  0.98 (0.97-0.99) |
| **P2P** | 1.00  0.99 (0.99-0.99) | 0.99  0.97 (0.95-0.98) | 1.00  1.00 (1.00-1.00) | 1.00  1.00 (1.00-1.00) | 0.99  0.99 (0.98-0.99) | 0.99  0.98 (0.97-0.99) |
| **Hindlimb-MEP** |  |  |  |  |  |  |
| **Latency** | 0.90  0.83 (0.75-0.88) | 0.35  0.13 (-0.05-0.32) | 0.97  0.94 (0.83-0.98) | 0.95  0.91 (0.78-0.97) | 0.99  0.98 (0.96-0.99) | 0.98  0.97 (0.94-0.98) |
| **AUC** | 0.98  0.95 (0.93-0.97) | 0.98  0.93 (0.78-0.97) | 0.99  0.99 (0.97-0.99) | 0.99  0.98 (0.96-0.99) | 0.96  0.93 (0.88-0.95) | 0.94  0.88 (0.80-0.93) |
| **P2P** | 1.00  1.00 (1.00-1.00) | 1.00  1.00 (0.99-1.00) | 1.00  1.00 (1.00-1.00) | 1.00  1.00 (1.00-1.00) | 0.98  0.96 (0.94-0.98) | 0.97  0.95 (0.91-0.97) |
| **SEP** | **N=12** | | **N=6** | | **N=4** | |
| **Forelimb** | 1  p=1.0 | 1  NA | 1  p=1.0 | 1  NA | 1  p=1.0 | 1  NA |
| **Hindlimb** | 1  p=1.0 | 1  NA | 1  p=1.0 | 1  NA | 1  p=1.0 | 1  NA |
| **Histology** |  |  |  |  |  |  |
| **Spinal cord histology** | **N=6** |  | **N=6** |  | **N=6** |  |
| **Adhesions** | 1  p=1.0 | 1  NA | 1  p=1.0 | 1  NA | 1  p=1.0 | 1  NA |
| **Thickness of tissue coverage** |  |  |  |  |  |  |
| **Left side** | 0.98  0.96 (0.92-0.98) | 0.97  0.94 (0.88-0.97) | 1.00  1.00 (0.99-1.00) | 0.98  0.97 (0.91-0.99) | 0.99  0.99 (0.97-1.00) | 0.75  0.54 (0.06-0.82) |
| **Middle** | 0.99  0.97 (0.95-0.99) | 0.97  0.94 (0.89-0.97) | 1.00  1.00 (0.99-1.00) | 0.84  0.71 (0.32-0.89) | 1.00  0.99 (0.98-1.00) | 0.90  0.76 (0.35-0.92) |
| **Right side** | 0.99  0.98 (0.96-0.99) | 0.98  0.96 (0.93-0.98) | 1.00  0.99 (0.98-1.00) | 0.86  0.75 (0.40-0.91) | 1.00  1.00 (0.99-1.00) | 0.87  0.76 (0.39-0.92) |
| **Neuronal cells (b3t) area (pixels)** | 1  1 (NA) | 1  1 (NA) | 1  1 (NA) | 1  1 (NA) | 1  1 (NA) | 1  1 (NA) |
| **Astroglial cells (GFAP) area (pixels)** | 1  1 (NA) | 1  1 (NA) | 1  1 (NA) | 1  1 (NA) | 1  1 (NA) | 1  1 (NA) |
| **Myelin (MBP) area (pixels)** | 1  1 (NA) | 1  1 (NA) | 1  1 (NA) | 1  1 (NA) | 1  1 (NA) | 1  1 (NA) |
| **Brain histology** | **N=6** |  | **N=4** |  | **N=5** |  |
| **Prefrontal cortex (neurons/mm2)** | 1  1 (NA) | NA | 1  1 (NA) | NA | 1  1 (NA) | NA |
| **Parietal cortex**  **(neurons/mm2)** | 1  1 (NA) | NA | 1  1 (NA) | NA | 1  1 (NA) | NA |
| **Hippocampus region CA1 (neurons/mm2)** | 1  1 (NA) | NA | 1  1 (NA) | NA | 1  1 (NA) | NA |
| **Caudate nucleus**  **(neurons/mm2)** | 1  1 (NA) | NA | 1  1 (NA) | NA | 1  1 (NA) | NA |
| **Thalamus (neurons/mm2)** | 1  1 (NA) | NA | 1  1 (NA) | NA | 1  1 (NA) | NA |
| **Corpus callosum**  **(neurons/mm2)** | 1  1 (NA) | NA | 1  1 (NA) | NA | 1  1 (NA) | NA |
| **Hindlimb muscle histology** | **N=6** |  | **N=6** |  | **N=6** |  |
| **Tensor fasciae latae muscle** | 0.92  p=1 | 0.778  NA | 1  p=1 | 1  NA | 0.875  p=1 | 0.875  NA |
| **Bladder and rectum histologic analysis** |  | All groups combined due to the small number of samples of good quality  Normal (n=2), nonM (n=1), myeloT (n=1) | | | | |
|  | **Intra-α**  **Intra-ICC (CI)** | | | **Inter-α**  **Inter-ICC (CI)** | | |
| **Bladder muscle thickness** | 0.81  0.70 (0.04-0.93) | | | 0.80  0.69 (0.03-0.93) | | |
| **Rectum muscle thickness**  **- Circular layer**  **- Longitudinal layer** | 0.95  0.90 (0.73-0.97)  0.95  0.88 (0.67-0.96) | | | 0.95  0.90 (0.73-0.97)  0.95  0.91 (0.74-0.97) | | |

**Table S3 – Magnetic Resonance Imaging (MRI) settings.**

*Abbreviations: TSE (Turbo Spin Echo); TIRM (Turbo Inversion Recovery Magnitude); HASTE (Half-Fourier Acquisition Single-shot Turbo spin Echo imaging); VIBE (Volumetric Interpolated Breath-hold Examination); Sag, Sagittal; Tra, Transversal; Cor, Coronal; FoV, Field-of-View; TR/TE, repetition time/echo time.*

| **Parameters** | **Sequence and weighing** | **planes** | **number of slices** | **Acquisition duration (min)** | **Base resolution** | **thickness (mm)** | **Intersection gap** | **FoV (mm)** | **TR/TE (ms)** |
| --- | --- | --- | --- | --- | --- | --- | --- | --- | --- |
| **Localization** |  |  |  |  |  |  |  |  |  |
| **Brain MRI**   - **Brain** - **Brainstem** - **Cervical spinal cord** | T1-TSE | Sag | 31 | 2:46 | 256 | 4 | 30% | 220 | 800/24 |
|  | T2-TSE | Sag  Tra  Cor | 22  22  30 | 3:07  2:38  1:26 | 448  320  320 | 3 | 0% | 220 | 6000/91 |
|  | T2-TIRM | Sag  Tra | 16  19 | 3:49  4:32 | 256  256 | 4 | 30% | 220 | 9000/95 |
|  | T2-HASTE | Tra | 8 | 0:16 | 256 | 4 | 20% | 350 | 2000/97 |
| **Spinal cord MRI**   - **Thorax (T9-T13)** - **Lumbar region** - **Sacro-coccygeal and tail** | T2-TSE | Sag | 13 | 2:01 | 320 | 3 | 10% | 300 | 3500/110 |
|  | T2-TIRM | Sag  Tra | 15  36 | 3:36  3:07 | 256  256 | 3  4 | 10% | 220 | 3700/47 |
| **Urinary tract MRI (from T9 to tail)** | T2-TIRM | Sag  Tra | 16  19 | 3:49  4:32 | 256  256 | 4 | 30% | 220 | 9000/95 |
| **Hindlimb muscles MRI** | T1-TSE | Sag | 31 | 2:46 | 256 | 4 | 30% | 220 | 800/24 |
|  | T1-VIBE | Tra  Cor | 144  128 | 2:58  2:38 | 384  256 | 2 | 20% | 400  420 | 8/3.690  2.97/1.03 |

Table S4 – Correlation analysis between motor function outcome measurements of the non-myelotomy and the myelotomy groups. We computed non-parametric Spearman and parametric Pearson correlation coefficients of 6 outcomes for 2 groups respectively: joint movement score, locomotor grade, MEPs area-under-the-curve (AUC) and peak-to-peak (P2P), area of spinal cord beta-III tubulin (b3T) on immunohistochemistry and hindbrain herniation distance (HH) on MRI. *Significant results are highlighted using asterisks as follows: *0.05 ≥ p > 0.01; **0.01 ≥ p > 0.001; ***0.001 ≥ p > 0.0001; ****p ≤ 0.0001.*

*Abbreviations: MEP, motor evoked potentials; MRI, magnetic resonance imaging; NA, non-applicable.*

|  | **Joint score** | **Locomotor grade** | **AUC** | **P2P** | **b3T area** | **HH** |
| --- | --- | --- | --- | --- | --- | --- |
| **Non-myelotomy** | **Spearman correlation coefficient** | | | | | |
| **Joint score** | NA | **1*** | NA | NA | -0.40 | -0.10 |
| **Locomotor grade** | **1*** | NA | NA | NA | -0.40 | -0.10 |
| **AUC** | NA | NA | NA | NA | NA | NA |
| **P2P** | NA | NA | NA | NA | NA | NA |
| **b3T area** | -0.40 | -0.40 | NA | NA | NA | -0.90 |
| **HH** | -0.10 | -0.10 | NA | NA | -0.90 | NA |
| **Myelotomy** | **Pearson correlation coefficient** | | | | | |
| **Joint score** | NA | **0.98**** | 0.86 | **0.92*** | -0.22 | -0.04 |
| **Locomotor grade** | **0.98**** | NA | 0.83 | **0.88*** | -0.15 | -0.05 |
| **AUC** | 0.86 | 0.83 | NA | **0.97**** | -0.50 | -0.25 |
| **P2P** | **0.92*** | **0.88*** | **0.97**** | NA | -0.45 | -0.00 |
| **b3T area** | -0.22 | -0.15 | -0.55 | -0.45 | NA | 0.00 |
| **HH** | -0.04 | -0.05 | -0.25 | -0.00 | 0.00 | NA |
